# Cor triatriatum sinister: a rare underlying cause of pulmonary hemosiderosis

**DOI:** 10.1186/s40064-016-1752-1

**Published:** 2016-02-24

**Authors:** Shuhan Peng, Yunbin Xiao, Jinwen Luo, Renwei Chen, Peng Huang, Pingbo Liu, Xicheng Deng

**Affiliations:** Department of Cardiothoracic Surgery, Hunan Children’s Hospital, No. 86 Ziyuan Road, Changsha, 410007 Hunan China; Department of Cardiology, Hunan Children’s Hospital, Changsha, 410007 Hunan China; Medical School, University of South China, Hengyang, China

**Keywords:** Cor triatriatum, Pulmonary hemosiderosis, Hemoptysis, Surgery

## Abstract

Pulmonary hemosiderosis is a disorder with unknown cause and characterized by hemosiderin appreciation in alveolar interstitium from decomposed hemoglobin following alveolar capillary bleeding, which finally leads to pulmonary fibrosis. It can be divided into primary and secondary types in terms of its etiology. While primary types are related to autoimmunity, secondary types can be associated with cardiovascular and pulmonary causes such as mitral stenosis leading to pulmonary congestion. We report a case of cor triatriatum sinister in a child who presented with hemoptysis as a main clinical manifestation and had been previously diagnosed with idiopathic pulmonary hemosiderosis. Based on clinical signs and imaging examinations, we considered the hemoptysis was most likely due to cor triatriatum. The child underwent corrective surgery with uneventful recovery. The hemoptysis has not recurred any more after operation. Cardiovascular disease including cor triatriatum should be considered with regards to the etiology of pulmonary hemosiderosis.

## Background

Pulmonary hemosiderosis is a disorder characterized by precipitation of hemosiderin from decomposed hemoglobin following alveolar capillary bleeding in alveolar interstitium (Ioachimescu et al. 2004). It can be divided into primary and secondary types. The latter can be associated with mitral stenosis that leads to pulmonary congestion (Ioachimescu et al. 2004; Datey and Kelkar 1970). Cor triatriatum is a rare congenital cardiac malformation (Chen et al. 1999). The combination of these two conditions is extremely rare. The case we report is a child with cor triatriatum in whom hemoptysis presented as a main clinical manifestation and idiopathic pulmonary hemosiderosis (IPH) was initially diagnosed. After detailed and thorough tests and examinations, we considered the hemoptysis was most likely due to cor triatriatum. The 4-year-old girl then had a corrective surgery for cor triatriatum, and has not had episodes of hemoptysis thereafter.

## Case presentation

**Fig. 1:**
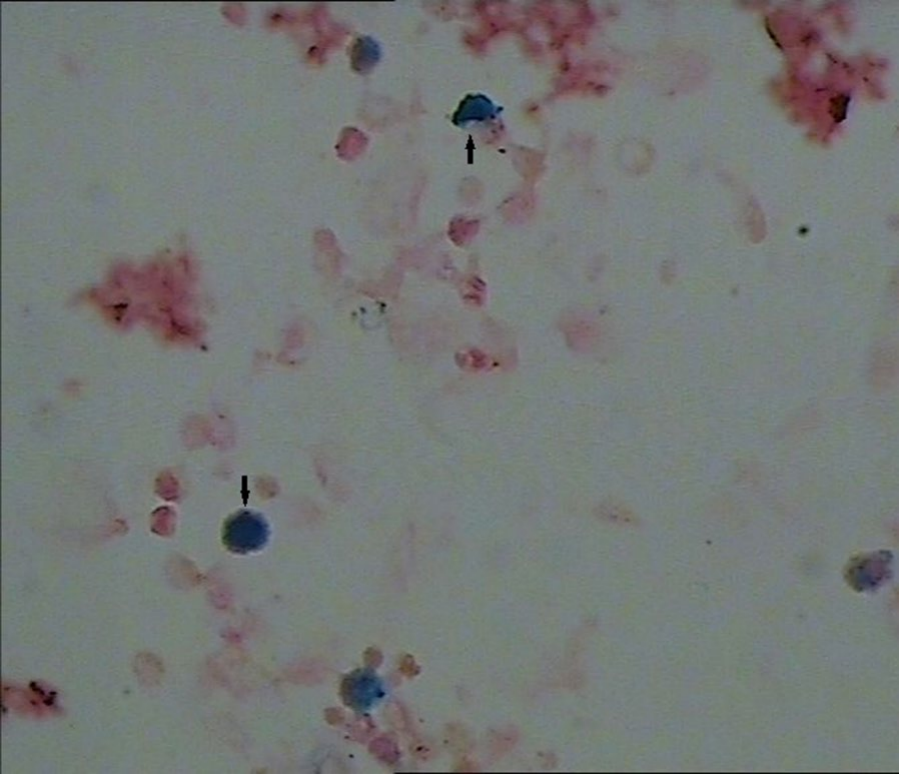
A small amount of iron staining particles and multiple hemosiderin phagocytic cells (*black arrows*) in sputum smear (iron staining + HE staining) is seen under microscope

**Fig. 2:**
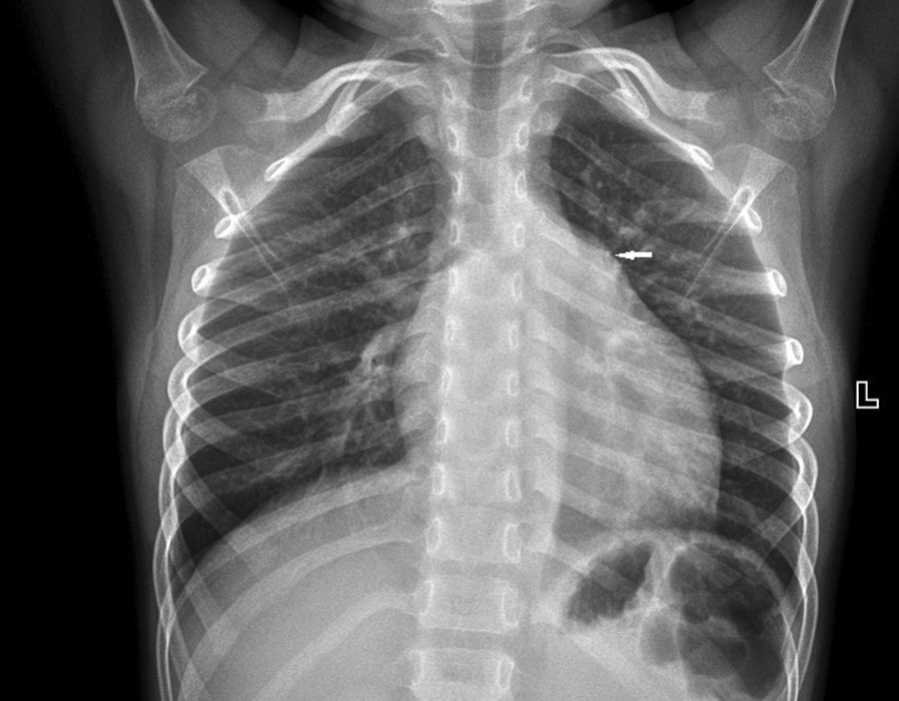
Bilateral lung markings thickening and prominent pulmonary artery segment (*white arrow*)

**Fig. 3:**
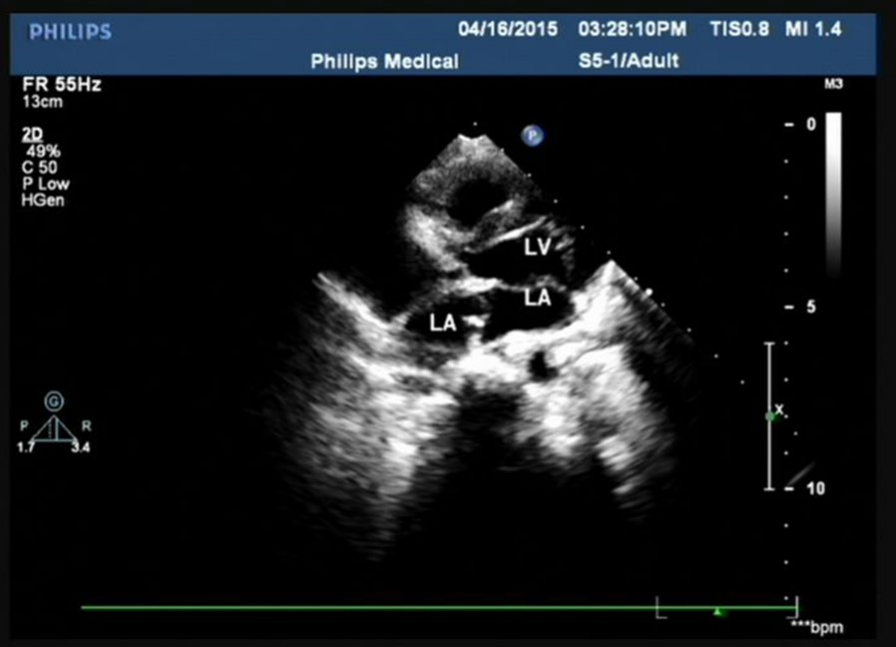
A 15 mm long high density band dividing the left atrium into true chamber and accessory chamber, with a 4-mm orifice connecting two chambers

**Fig. 4:**
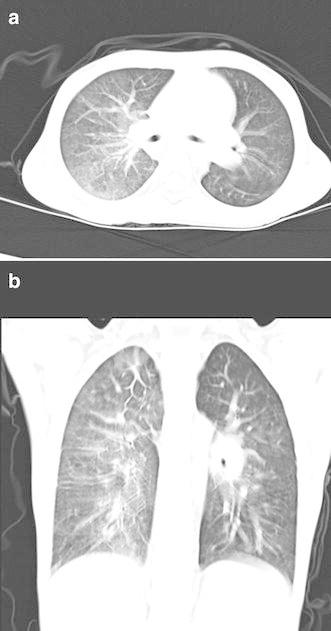
**a** The cross section of CT shows bilaterally increased pulmonary vasculature, especially in the right side. **b** The coronal plane shows bilaterally increased pulmonary vasculature, especially in the right side

**Fig. 5:**
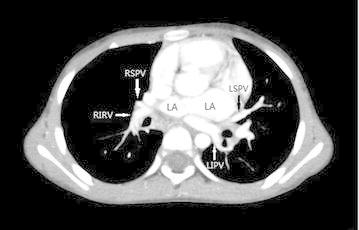
The left atrium’s division is visible, as well as left pulmonary veins draining into true chamber, right pulmonary veins draining into accessory chamber

**Fig. 6:**
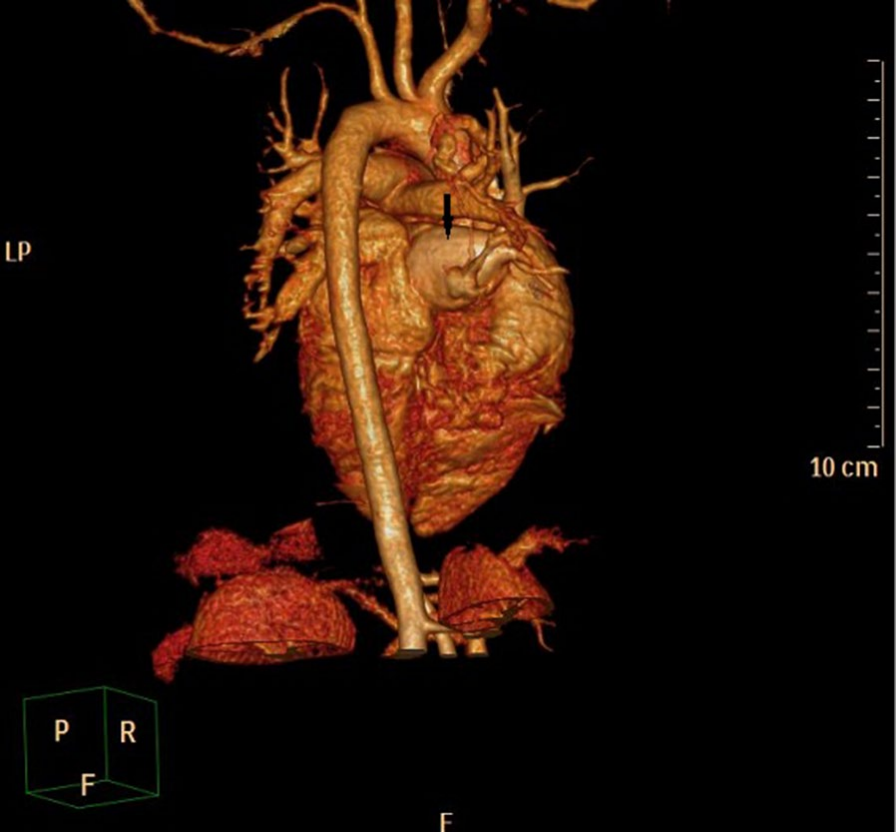
Right pulmonary venous confluence distention (*black arrow*)

A formal written consent has been obtained from the parents regarding publication of the following child’s personal and medical information. A 4-year-old girl was recently hospitalized for a heart murmur found 4 years ago. Physical examination revealed coarse lung breath sounds and a II/VI rough systolic murmur on the left sternal border at 2–3 intercostal space. She had 3 episodes of hemoptysis in total over the last 6 months and two visits to respiratory medicine clinic 2 and 3 months ago, respectively. During the visits, sputum was sampled from lower respiratory tract using the bronchial microscope. A few iron staining particles and multiple hemosiderin phagocytic cells in sputum smear (iron staining + HE staining) were found under microscopy (Fig. 1). She was diagnosed as “idiopathic pulmonary hemosiderosis disease” and treated with methylprednisolone 10 mg/day. The doses were gradually reduced month by month. As such, she had been on methylprednisolone for four consecutive months before this admission. She was anemic 4 months ago before initiation of corticosteroids (Hbs 87–94 g/L), During present hospitalization, the preoperative Hb was 118 g/L, oxygen saturation 96 %. Her chest X-ray showed thickened bilateral upper lung markings and a prominent pulmonary artery segment (Fig. 2). Echocardiography showed a 15 mm-long high density band dividing the left atrium into a true chamber and a accessory chamber, with a 4-mm orifice connecting the two chambers (Fig. 3). The left pulmonary veins drained into true chamber, yet the right pulmonary veins drained into accessory chamber with prominent local distention of pulmonary veins. Atrial septum was visible. The pressure gradient (PG) across the orifice, the diastolic trans-pulmonary PG and the systolic trans-tricuspid PG using Doppler were 58, 52, 107 mmHg, respectively, suggesting severe stenosis across the orifice and pulmonary hypertension. A computerized tomography (CT) scan of the chest showed bilaterally increased pulmonary vasculature, especially in the right side (Fig. 4a, b). A three-dimensional (3D) reconstruction revealed the division in the left atrium, left pulmonary veins draining into the true chamber, yet right pulmonary veins draining into the accessory chamber (Fig. 5) with considerable distention of pulmonary venous confluence (Fig. 6). Since she had been on corticosteroid for 4 months, the risk of postoperative wound dehiscence and infection was considered high. However, with a high index of suspicion that the hemoptysis was from pulmonary congestion secondary to cor triatriatum, we proceeded with a corrective operation. She was operated on under cardiopulmonary bypass. The atrial septum was divided. Our exploration and findings confirmed preoperative diagnosis. A complete removal the membrane between accessory and true chambers was carried out to make sure blood flow was not restricted. The postoperative recovery was uneventful, and she was discharged on postoperative day 5. Eight days after discharge, she was re-hospitalized due to the upper wound dehiscence for secondary suture. She recovered well and was discharged again. There was no further hemoptysis at 6 months follow-up.

## Discussion

Cor triatriatum is a rare congenital cardiac malformation, the incidence of which has been reported as 0.1–0.4 % of all congenital heart diseases (Chen et al. 1999). It is classified into cor triatriatum sinister and cor triatriatum dexter, with the latter one rarer (Krasemann et al. 2007). Cor triatriatum sinister is characterized by an abnormal separation of the left atrium into a pulmonary venous chamber and a true chamber (Richardson et al. 1981). It can occur as an isolated anomaly in approximately 30 % of cases; however, it is frequently associated with other cardiac anomalies, such as patent ductus arteriosus, atrial septal defect, anomalous pulmonary venous drainage, and persistent left superior vena cava (Chen et al. 1999; Alphonso et al. 2005). Although symptoms usually present in infancy, cor triatriatum sinister can initially occur in adulthood when the membrane contains large fenestration or other drainage exists (Frogel and Galusca 2010). Several classifications have been developed. According to the Lucas classification, it can be classified into three types. Type I means all four veins drain into the accessory chamber, which in turn communicates with the true chamber. Subtyping is based on the presence or absence of other connections with the accessory chamber. Type II indicates the accessory chamber receives all four pulmonary veins but does not communicate with the left atrium. Type III includes the rest, with various subtypes according to vein connections that do not connect to the accessory chamber. In this case, the child’s left pulmonary veins drained into the true chamber, the right pulmonary veins drained into the accessory chamber. As per literature we have tried to find out, it’s the first Type III cor triatriatum sinister case complicated with pulmonary hemosiderosis. The hemodynamics depends upon the size and number of openings in the membrane and may vary from normal to similar to mitral stenosis (Alphonso et al. 2005). Patients with fenestrations more than 1 cm in diameter are usually asymptomatic (Yoo et al. 2009; Penafiel and Yeo 2008). There is a case report that a child with a 6 mm orifice who did not presented with clinical symptoms until 8-year-old (Sritippayawan et al. 2002). While in our patient the opening was only 4 mm. The small size of opening is extrapolated to be the reason of early presentation. Cor triatriatum sinister produces symptoms by causing pulmonary venous obstruction and pressure overload in the right heart (Lin et al. 2005). The most frequent initial symptoms in infants are respiratory distress, cyanosis, recurrent respiratory tract infections and feeding difficulties, while older patients may present with syncope, dyspnea and hemoptysis (Chen et al. 1999; Lin et al. 2005). Echocardiography is the first-line examination for diagnosis of cor triatriatum (Su et al. 2008).

Pulmonary hemosiderosis can be divided into primary and secondary types (Ioachimescu et al. 2004). Idiopathic pulmonary hemosiderosis is a rare cause of diffuse alveolar hemorrhage of unknown etiology (Ioachimescu et al. 2004). It occurs most frequently in children (Saeed et al. 1999). The etiology of IPH may include autoimmunity, genetic factors, environment, allergy, etc. (Ioachimescu et al. 2004). Patients often present with diffuse alveolar hemorrhage, which is characterized by dyspnea and hemoptysis, diffuse and bilateral alveolar infiltrates on chest radiograph and anemia (Green et al. 1996). There is no pathognomonic finding for IPH but a few radiological patterns closely relate to clinical phases. During the acute phase (IPH exacerbations) the chest radiographs show diffuse alveolar-type infiltrates, predominantly in the lower lung fields (Ioachimescu et al. 2004). Clinical symptoms may include cough, pneumonia, hypoxia and anemia whereas confirmative diagnosis is based on the presence of hemosiderin-laden macrophages without any evidence of pulmonary vasculitis, nonspecific granulomatous inflammation or deposition of immunoglobulins in respiratory secretion or biopsy materials (Ioachimescu et al. 2004). Secondary pulmonary hemosiderosis is with similar clinical symptoms, but can be tracked with a specific cause, such as pulmonary vascular malformations, coagulation disorders, systemic lupus erythematosus, mitral stenosis, etc. (Ioachimescu et al. 2004). A study (Salih et al. 2006) suggests a cutoff value of hemosiderin-laden macrophage index as a lab diagnostic criterion for IPH, yet its efficacy has not been verified in secondary pulmonary hemosiderosis. With regard to the causes, rheumatic mitral stenosis is more common as a secondary cause, and can result in lung congestion, pulmonary hypertension and then pulmonary hemosiderosis (Agrawal et al. 2011). Cor triatriatum sinister, as previously mentioned, may cause similar pathophysiological changes to that of mitral stenosis. It can also lead to pulmonary hemosiderosis. In view of this case’s preexisting congenital heart disease, Consideration of a secondary type hemosiderosis should have been given in this child’s diagnosis. Unfortunately, when the girl was treated in respiratory clinic, although having acknowledged her congenital heart disease history, her doctor failed to take it into account.

For idiopathic pulmonary hemosiderosis, systemic glucocorticoids can reduce the morbidity and mortality from acute episodes of alveolar bleeding and control progression to pulmonary fibrosis (Green et al. 1996; Kiper et al. 1999). The recommended dose is <1 mg/kg/day prednisolone for 2 months and then gradually reduce it (Gencer et al. 2007). And there are also some studies suggesting 2–5 mg/kg/day prednisone (Chryssanthopoulos et al. 1983; Kjellman et al. 1984). Our patient started methylprednisolone 4 months ago, with an initial dose of 20 mg/day, which was then reduced month by month. Before the surgery, the dose was 5 mg/day. The echocardiography showed the communication between the accessory and true chambers was narrow when she came to our department for treatment, with a PG of 58 mmHg. With the aid of CT scan and 3D reconstruction, we diagnosed her as cor triatriatum sinister with a severe stenosis. The CT showed the lungs had congestion presentation on both sides, but it was more prominent on the right side, which was consistent with its pulmonary vein connections. Surgical treatment is the only way to correct cor triatriatum. In view of cor triatriatum as the patient’s probable cause of hemosiderosis, we operated on her under cardiopulmonary bypass. Removal the membrane between accessory and true chambers was complete to make blood flow smooth and unlimited. Complete resection of the membrane without injuring the atrial wall is the key to successful operation. There has been no further hemoptysis over 6 months follow-up. And we believe this is because pulmonary hypertension has been corrected through repair cor triatriatum and the potential mechanism which resulted in hemosiderosis has been eliminated.

## Conclusions

We report a very rare combination of pulmonary hemosiderosis with cor triatriatum sinister. When primary pulmonary hemosiderosis is considered, precautions should be given to exclude all possible underlying causes before a diagnosis is achieved. In such a case as presented here, hemosiderosis can be well managed by correction of the causative factor.

## Abbreviations

IPH: idiopathic pulmonary hemosiderosis
CT: computerized tomography
3D: three-dimensional
Hb: hemoglobin
PG: pressure gradient
